# Effectiveness of AI-based conversational and socially assistive agents in older adults: a systematic review and meta-analysis

**DOI:** 10.1186/s12877-026-07418-6

**Published:** 2026-05-07

**Authors:** Wenling Gou, Florian Lefebvre, Tongping Yang, Robin Recours, Jing Yang

**Affiliations:** 1 Santesih UR_UM211, University of Montpellier, Montpellier, France; 2 Hanoi School of Business and Management, Vietnam National University, Hanoi, Vietnam; 3 School of Education Science, Chengdu Normal University, Chengdu, China; 4 School of Psychology and Sociology, Mianyang normal university, Mianyang, China

**Keywords:** Artificial intelligence, Older adults, Depression, Loneliness, Psychological chatbots

## Abstract

**Background:**

Depression and loneliness are highly prevalent among older adults, yet access to timely and adequate mental health care remains limited in this population. Artificial intelligence–based conversational and socially assistive agents have emerged as a potentially scalable and cost-effective intervention; however, their effectiveness in alleviating depression and loneliness among older adults has not been comprehensively established. This systematic review and meta-analysis aimed to synthesize evidence from randomized controlled trials (RCTs) examining the effects of AI-based conversational and socially assistive agent interventions on depressive symptoms and loneliness in older adults.

**Methods:**

A systematic search of five electronic databases was conducted from inception to November 15, 2025, to identify RCTs evaluating AI-based conversational and socially assistive agent interventions targeting depression and/or loneliness in older adults. Random-effects meta-analyses were performed using standardized mean differences. Statistical heterogeneity was assessed using the I² statistic and further explored through subgroup analyses. Risk of bias was evaluated using the Cochrane Risk of Bias 2 tool, and the certainty of evidence was appraised using the GRADE framework.

**Results:**

Eight RCTs comprising 611 participants met the inclusion criteria. Compared with control conditions, AI-based conversational and socially assistive agent interventions were associated with a statistically significant reduction in depressive symptoms (Hedges’ g = − 0.25, 95% CI − 0.48 to − 0.02; I² = 10.7%). In contrast, no significant effect was observed for loneliness, and substantial heterogeneity was detected across studies (Hedges’ g = − 0.67, 95% CI − 2.57 to 1.23; I² = 89%). Subgroup analyses suggested that interventions with a cognitive focus yielded more consistent effects than companionship-focused approaches, while no clear differences were observed between home-based and institutional settings.

**Conclusions:**

AI-based conversational and socially assistive agent interventions appear to be effective in reducing depressive symptoms among older adults, whereas current evidence does not support a significant effect on loneliness. The effectiveness of these interventions may depend on their theoretical orientation and implementation characteristics. AI-based conversational and socially assistive agents may serve as a promising adjunct to conventional mental health care for older adults; however, further high-quality trials are needed to clarify their role in addressing loneliness and to optimize intervention design.

**Protocol registration:**

The protocol for this systematic review was registered in International Prospective Register of Systematic Reviews (PROSPERO identifier: CRD420261283098).

**Supplementary Information:**

The online version contains supplementary material available at 10.1186/s12877-026-07418-6.

## Background

Mental health is a fundamental component of overall well-being and daily functioning. With the rapid aging of the global population, however, older adults are facing increasingly pronounced mental health challenges [1]. Accumulating evidence indicates that the prevalence of depression and anxiety among older adults continues to rise, with substantial adverse consequences for quality of life, social functioning, and physical health [2]. Despite the growing demand for mental health support, timely access to appropriate care remains limited for many older adults due to multiple structural and individual barriers, including shortages of mental health professionals, mobility constraints, and restricted accessibility of services [3]. Concurrently, conventional interventions such as pharmacotherapy, face-to-face psychotherapy, and community-based programs present notable limitations in older populations, including the risk of adverse side effects, suboptimal treatment adherence, and limited scalability [4]. Together, these challenges highlight an urgent need to develop innovative, cost-effective, and accessible mental health interventions that are better aligned with the specific needs and circumstances of older adults [5].

In recent years, digital mental health interventions have gained increasing attention as promising approaches to addressing gaps in mental health service delivery for older adults, largely attributable to their scalability, relatively low cost, and improved accessibility [6, 7]. Within this domain, artificial intelligence-driven conversational and socially assistive agents have emerged as a particularly promising modality. These systems typically employ natural language processing technologies to simulate human-like conversations through text or voice-based interfaces, or may be embedded within socially assistive robotic platforms to facilitate interactive engagement, thereby delivering real-time emotional support, psychoeducation, cognitive restructuring, and problem-solving guidance [8]. Moreover, some AI-based systems incorporate guided relaxation techniques, cognitive behavioral strategies, and emotion regulation exercises, thereby targeting maladaptive cognition, enhancing coping capacity, and supporting emotional self-regulation [9, 10]. Owing to their anonymity, low threshold for engagement, and independence from temporal and geographic constraints, AI-driven conversational and socially assistive agents may be particularly well suited for older adults who experience mental health difficulties but encounter barriers to accessing traditional services [11].

Recent empirical studies have begun to investigate the potential effectiveness of AI-based conversational and socially assistive agents in improving psychological outcomes among older adults. For instance, a study conducted during the COVID-19 pandemic found that AI-based agent interventions were associated with reductions in mild to moderate depressive symptoms and loneliness among older adults, thereby contributing to improved emotional well-being [12]. Despite these encouraging findings, significant challenges remain in this rapidly developing field. The existing evidence base is predominantly derived from studies with small sample sizes, a limited number of randomized controlled trials, and substantial heterogeneity with respect to system design, intervention content, cultural context, and the degree of human-computer interaction [13, 14]. Collectively, these limitations constrain the robustness and generalizability of current conclusions.

In light of these considerations, a systematic review and meta-analysis are warranted to comprehensively synthesize and quantitatively evaluate evidence from existing randomized controlled trials. This approach allows for a more precise estimation of the overall effects of AI-driven conversational and socially assistive agent interventions on depression and loneliness among older adults, together with the associated uncertainty. Furthermore, it facilitates examination of the potential influence of system characteristics, intervention modalities, and study design features on intervention outcomes, thereby supporting interpretation of between-study heterogeneity. Accordingly, the present study aims to systematically review and meta-analyze randomized controlled trials assessing the effectiveness of AI-based conversational and socially assistive agents in reducing depression and loneliness among older adults, to provide robust evidence to inform clinical decision-making and guide future research.

## Methods

### Protocol and registration

This review was conducted and reported in accordance with the PRISMA statement [15], with the protocol prospectively registered in PROSPERO (CRD420261283098).

### Eligibility criteria

Studies were included if they involved older adults receiving an artificial intelligence-based conversational or socially assistive agent intervention. Older adults were defined as individuals aged 60 years or older, or samples with a mean age of at least 60 years, and both male and female participants were considered. Interventions needed to utilize artificial intelligence technologies, such as natural language processing or machine learning, to provide psychological support, guidance, or interactive engagement through conversational agents, including AI-powered chatbots. Only interventions with explicit psychological or mental health objectives were considered, whereas non-AI approaches and purely rule-based systems without adaptive capabilities were excluded. Eligible studies were required to report at least one quantitative outcome related to depression or loneliness, measured using validated instruments. Outcomes of interest encompassed changes in depressive symptoms, loneliness levels, or both, assessed at post-intervention and, where available, at follow-up. Only randomized controlled trials that met the population, intervention, and outcome criteria were retained, with no restrictions on publication year or geographic region.

Studies were excluded if they focused on non-older adult populations, did not involve an AI-based conversational or socially assistive agent intervention, failed to report depression or loneliness outcomes separately, or employed a study design other than a randomized controlled trial, including observational studies, qualitative studies, case reports, protocols, reviews, or conference abstracts. For overlapping study samples, the report with the most comprehensive data or the longest follow-up was selected.

### Information sources and search strategy

A systematic literature search was conducted across PubMed, Embase, Web of Science, the Cochrane Library, and PsycArticles, covering all records published from database inception to November 15, 2025. The search strategy was organized around three core domains. The first domain comprised terms identifying the target population of older adults, including older adults, elderly, aging, and older people. The second domain included terms describing the intervention of interest, namely artificial intelligence-based conversational and socially assistive agents, including artificial intelligence, AI, chatbot, conversational agent, and mental health chatbot. The third domain encompassed terms related to depression- and loneliness-related psychological outcomes, including depression and loneliness, as well as closely related constructs frequently used to index these outcomes in prior research, such as depressive symptoms, social isolation, mental health, and emotional well-being.

To enhance the identification of high-quality evidence, search filters and keywords related to randomized controlled trials were applied, including randomized controlled trial and RCT. Search concepts were combined using Boolean operators (AND and OR), and database-specific controlled vocabulary terms, such as MeSH and Emtree, were incorporated when appropriate. Detailed search strategies for each database are provided in Supplementary File 1.

### Selection process

Following removal of duplicate records, two reviewers (W.G. and T.Y.) independently screened titles and abstracts to assess study eligibility. Articles deemed potentially eligible were subsequently subjected to full-text review in accordance with predefined inclusion and exclusion criteria. Any discrepancies arising during the study selection process were resolved through discussion and, when necessary, consultation with the corresponding reviewer (J.Y.), thereby ensuring methodological consistency and rigor.

### Data collection process and data items

Data from each included study were independently extracted by two reviewers (W.G. and T.Y.) using a standardized data extraction form. Collected information included first author and year of publication, country, study characteristics, sample size, participant characteristics such as mean age and gender distribution, intervention characteristics including type of AI-based conversational or socially assistive agent, intervention orientation, delivery setting, duration, and frequency, comparator details, and outcome measures related to depression or loneliness.

For quantitative synthesis, post-intervention mean values and standard deviations of depression and/or loneliness outcomes were extracted for both intervention and control groups. When multiple validated instruments were reported, data from the primary outcome measure specified by the study authors were preferentially used. If post-intervention standard deviations were not directly provided, they were calculated from other available statistical information, such as standard errors, confidence intervals, or p-values, using established methods. Any disagreements were resolved through discussion among reviewers, with unresolved cases adjudicated by J.Y.

### Risk of bias

The risk of bias in the included randomized controlled trials was independently assessed by two reviewers using the Cochrane Risk of Bias tool (RoB 2), which evaluates five domains: the randomization process, deviations from intended interventions, missing outcome data, outcome measurement, and selection of the reported result [16]. Any disagreements were resolved through discussion or consultation with a third reviewer.

### Certainty of the evidence

The certainty of the evidence was assessed using the GRADE approach. Evidence was downgraded based on factors such as risk of bias, inconsistency, indirectness, imprecision, or publication bias, and the overall certainty for each outcome was classified as high, moderate, low, or very low [17].

### Synthesis methods

Meta-analyses were conducted to synthesize the effects of artificial intelligence-based conversational and socially assistive agent interventions on depression and loneliness outcomes by comparing post-intervention scores between intervention and control groups. Effect sizes were calculated as standardized mean differences (SMDs; Hedges’ g) with corresponding 95% confidence intervals (CIs) to account for the use of different validated outcome measures across studies. A random-effects model was applied to account for anticipated heterogeneity among studies [18].

Between-study variance was estimated using the restricted maximum likelihood (REML) method, and the Hartung–Knapp adjustment was applied to derive more conservative confidence intervals under the random-effects framework [19]. Statistical heterogeneity was assessed using the I² statistic, with values of approximately 25%, 50%, and 75% indicating low, moderate, and high heterogeneity, respectively.

Subgroup analyses were conducted to explore potential sources of variability and to examine whether intervention effects differed according to intervention characteristics, including living environment and intervention orientation. Intervention orientation was defined according to the primary stated objective and core components of each intervention. Interventions explicitly targeting structured cognitive stimulation or cognitive training were classified as cognitive-focused, whereas interventions primarily designed to provide companionship, social engagement, or relational interaction were classified as companionship-focused. All statistical analyses were performed using R software (version 4.4.0; R Foundation for Statistical Computing, Vienna, Austria), employing the meta and metafor packages. A two-tailed p-value < 0.05 was considered statistically significant. Publication bias was not formally assessed using funnel plots or statistical tests because fewer than 10 studies were included in the meta-analysis, in line with methodological recommendations [20].

## Results

### Study selection

The database search strategy applied to PubMed, Web of Science, Embase, the Cochrane Library, and PsycArticles yielded 3,491 potentially relevant records. After the removal of 1,977 duplicate entries, 1,514 unique records remained for title and abstract screening, during which 1,450 records were excluded.

**Fig. 1:**
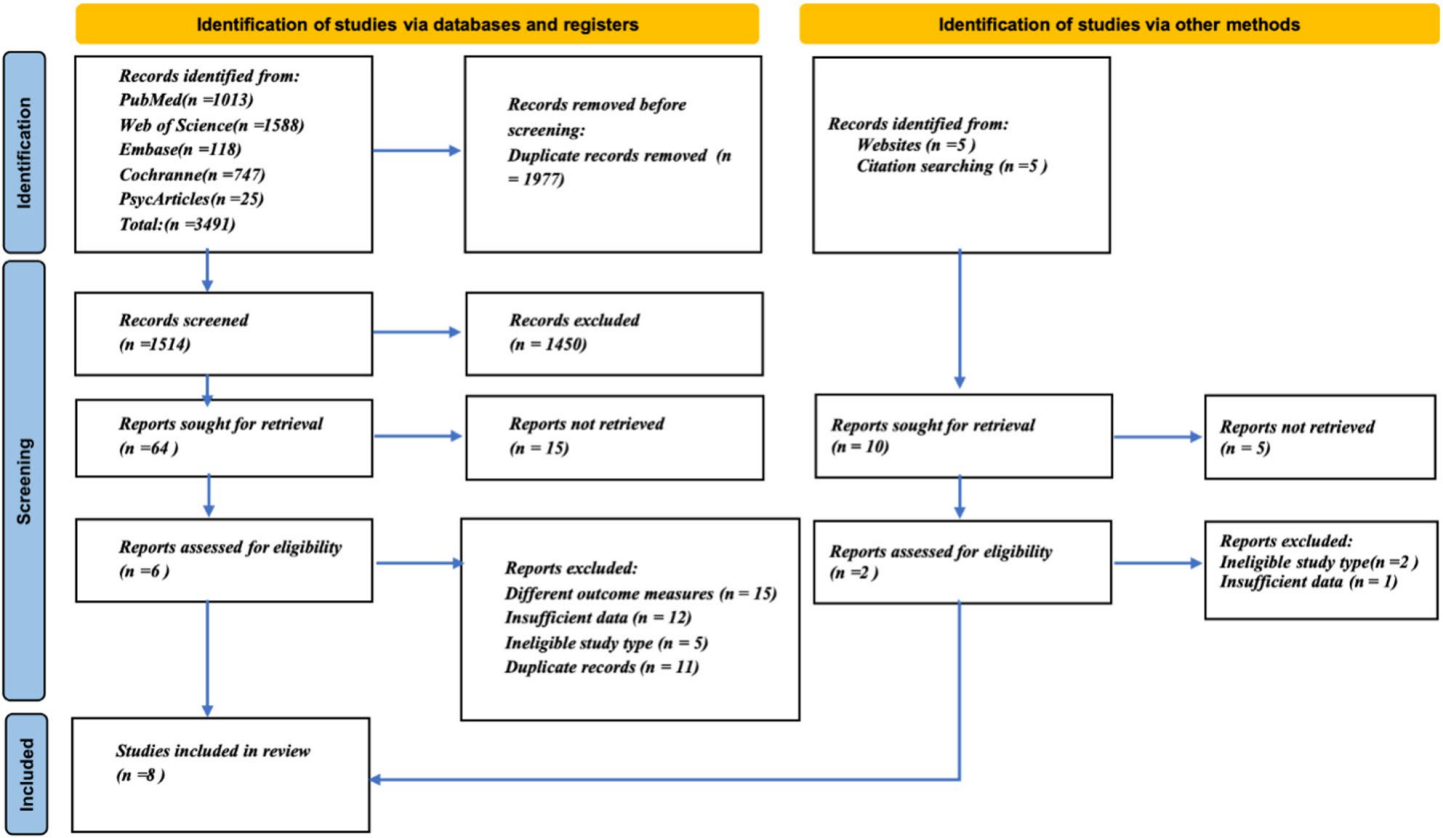
PRISMA 2020 flow diagram showing the process of study identification, screening, eligibility assessment, and inclusion

Full-text retrieval was attempted for 64 reports, of which 15 could not be obtained. The remaining 49 reports were assessed for eligibility, and 43 were excluded for reasons including irrelevant outcomes (*n* = 15), insufficient data for analysis (*n* = 12), inappropriate study design (*n* = 5), and duplicate publications (*n* = 11). As a result, 6 studies identified through database searches were included. In addition, two further articles identified through supplementary searches were screened for eligibility. Ultimately, 8 studies [13, 14, 21–26] were included in the systematic review and meta-analysis. The study selection process is illustrated in Fig. 1.

### Study characteristics

Across the 8 included studies, a total of 611 older adults from eight different countries were analyzed. The sample sizes of individual studies ranged from 24 to 142 participants. All included studies examined the effects of artificial intelligence-based or robot-assisted interventions on psychological outcomes in older adults, with depression and or loneliness specified as primary outcomes. Depression was most commonly assessed using the Geriatric Depression Scale Short Form or the Cornell Scale for Depression in Dementia, while loneliness was measured using the University of California, Los Angeles Loneliness Scale or its validated short form versions. The duration of interventions ranged from 2 to 12 weeks, with session frequencies varying from once weekly to daily use, and session lengths ranging from approximately 20 to 60 min.

The interventions encompassed robot-assisted cognitive training, computerized cognitive training applications, social or companion robots, and conversational voice assistant-enhanced routines and were delivered in both individual and group formats. Participants’ mean ages ranged from the mid-seventies to the mid-eighties across studies. Comprehensive characteristics of the included studies are presented in Table 1.

**Table 1:** Characteristics of included literature

Author	Country	*N* (total sample)	Measure	Duration frequency	% Female	Age(years)	Intervention	Reported AI Components
Park (2021) [21]	South Korea	135	Geriatric Depression Scale Short Form – Korean version	6 weeks; 12 sessions; 2/week; 60 min	E:71% C:73%	E: 75.5 ± 5.9 C: 75.6 ± 6.6	Robot-Assisted Cognitive Training	Humanoid robot with user-responsive interaction and emotion-related behaviors
Calenti (2015) [13]	Spain	142	Geriatric Depression Scale – Short Form	12 weeks, 2 sessions/week, ~ 20 min each	E:76.2% C:72.6%	E: 73.45 ± 5.95 C: 75.48 ± 6.85	Computerized cognitive training application	Interactive digital cognitive training system with structured task progression
Lim (2025) [22]	Korea	66	Geriatric Depression Scale – Short Form	6 weeks, 2 sessions/week, 50 min each	E:81.8% C:72.7%	E:82.9 ± 5.45 C:84.4 ± 4.94	Social robot program	Embodied social robot with interactive behavioral responses
Liang (2017) [23]	New Zealand	24	Cornell Scale for Depression in Dementia	6 weeks (day care: 2–3 times/week, 30-min/session; home: 6 weeks continuous use)	E:61.5% C:63.6%	E:84.6 ± 9.09 C:82.9 ± 6.72	Companion robot program	Companion robot providing multi-sensory interactive engagement
Shade (2025) [24]	United States	50	Geriatric Depression Scale Short Form University of California Los Angeles Loneliness Scale Version 3	12 weeks, twice a day + as needed	E:88% C:88%	E:79 ± 7.85 C:79 ± 7.85	Conversational robot	Conversational voice-based interactive system
Pu (2020) [25]	Australia	43	Cornell Scale for Depression in Dementia	6 weeks, 30-min individual nonfacilitated sessions 5 days/week	E:85.7% C:54.5%	E:86.48 ± 8.81 C:85.55 ± 5.80	Companion robot	Embodied companion robot with responsive interaction
Chen (2024) [26]	Taiwan	118	Geriatric Depression Scale-Short Form University of California Los Angeles Loneliness Scale-version 3	6 weeks, 30-min group sessions once/week	E:60.3% C:73.3%	E:81.78 ± 7.39 C:82.12 ± 7.12	Companion robot	Socially assistive companion robot with interactive engagement
Papadopoulos (2022) [14]	UK	33	Short Form UCLA Loneliness Scale	2 weeks, 6/week×3 h	E:21.2% C:24.2%	E:79.50 ± 9.03 C:84.90 ± 10.44	Socially Assistive Robots	Culturally competent socially assistive robot with AI-driven interaction

### Risk of bias

**Fig. 2:**
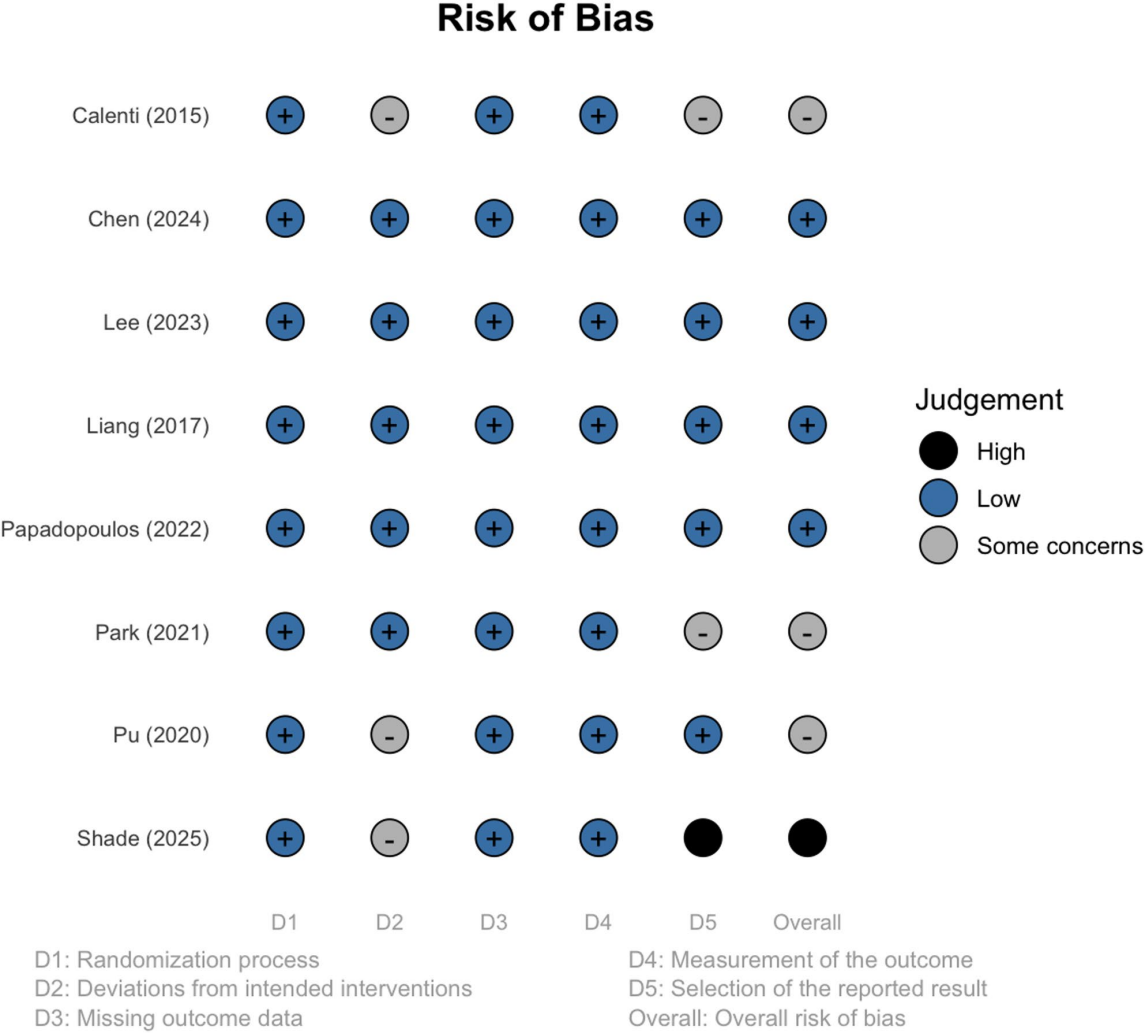
Risk of bias assessment based on the ROB-2 tool

Using the Cochrane Risk of Bias 2 (RoB 2) tool, the overall risk of bias among the included randomized controlled trials was evaluated. Most studies were judged to have a low risk of bias, although concerns were identified in specific domains in several studies, and one study was classified as having a high risk of bias (Fig. 2). Overall, the methodological quality was considered adequate for quantitative synthesis; however, the presence of residual bias necessitates cautious interpretation of the pooled findings.

### Certainty of evidence

**Fig. 3:**
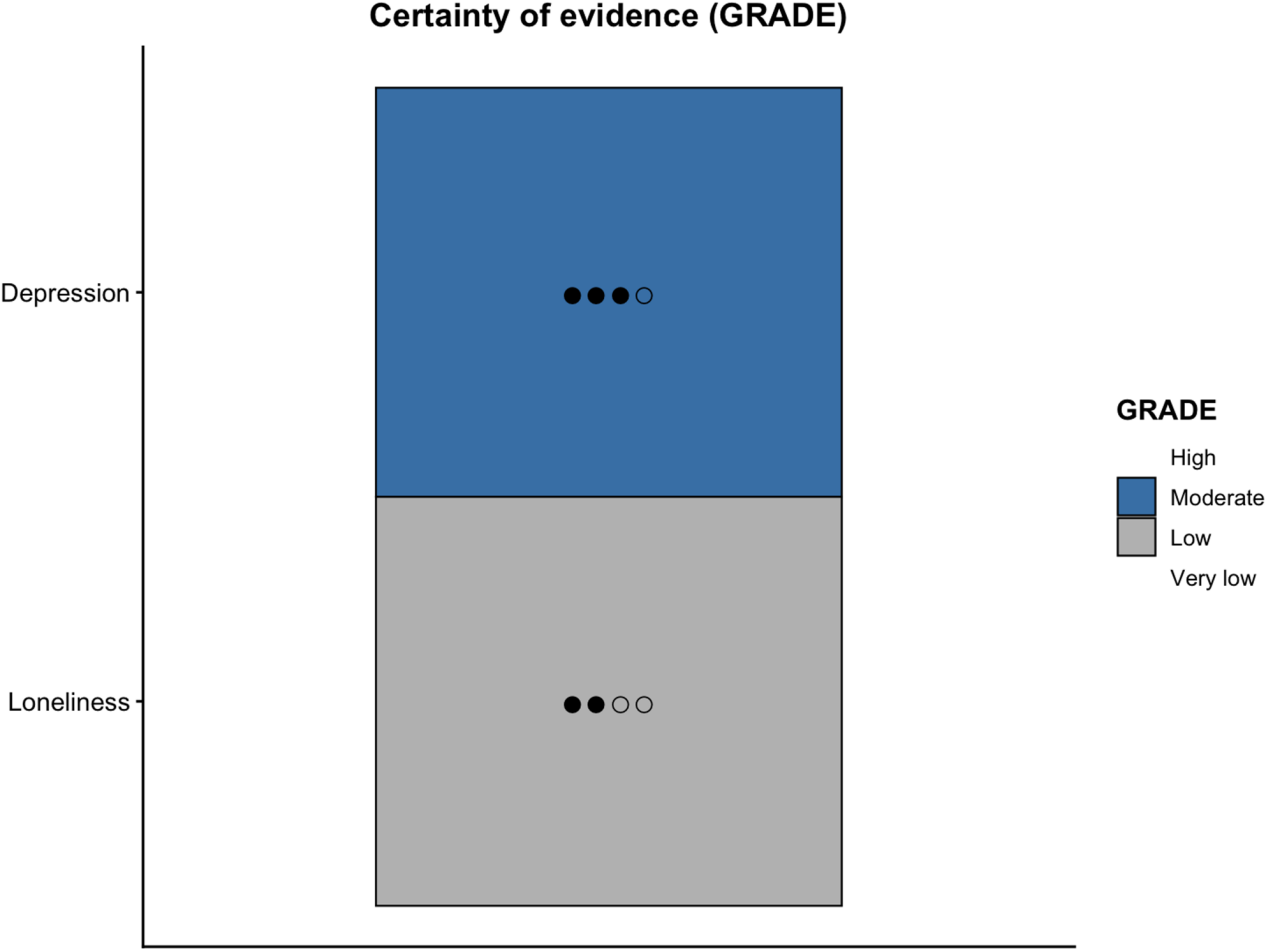
Certainty of evidence (GRADE) for depression and loneliness outcomes

According to the GRADE approach, the certainty of evidence was rated as moderate for depression and low for loneliness (Fig. 3). The downgrading of evidence was primarily due to concerns related to risk of bias and imprecision. These ratings indicate that the findings for depression are relatively robust, whereas the evidence for loneliness requires cautious interpretation.

### Sensitivity analysis

A leave-one-out sensitivity analysis was conducted using a random-effects model to evaluate the robustness of the pooled estimates (Supplementary Figures S1). For depressive outcomes, the pooled effect size remained consistent in direction across all iterations, with only minor variation in magnitude, and no single study materially altered the overall interpretation of the findings. For loneliness outcomes, although the exclusion of individual studies reduced heterogeneity estimates, the pooled effect direction remained unchanged and the overall results remained statistically non-significant. These findings indicate that the conclusions were not driven by any individual study.

### Results of syntheses

#### Depression

**Fig. 4:**
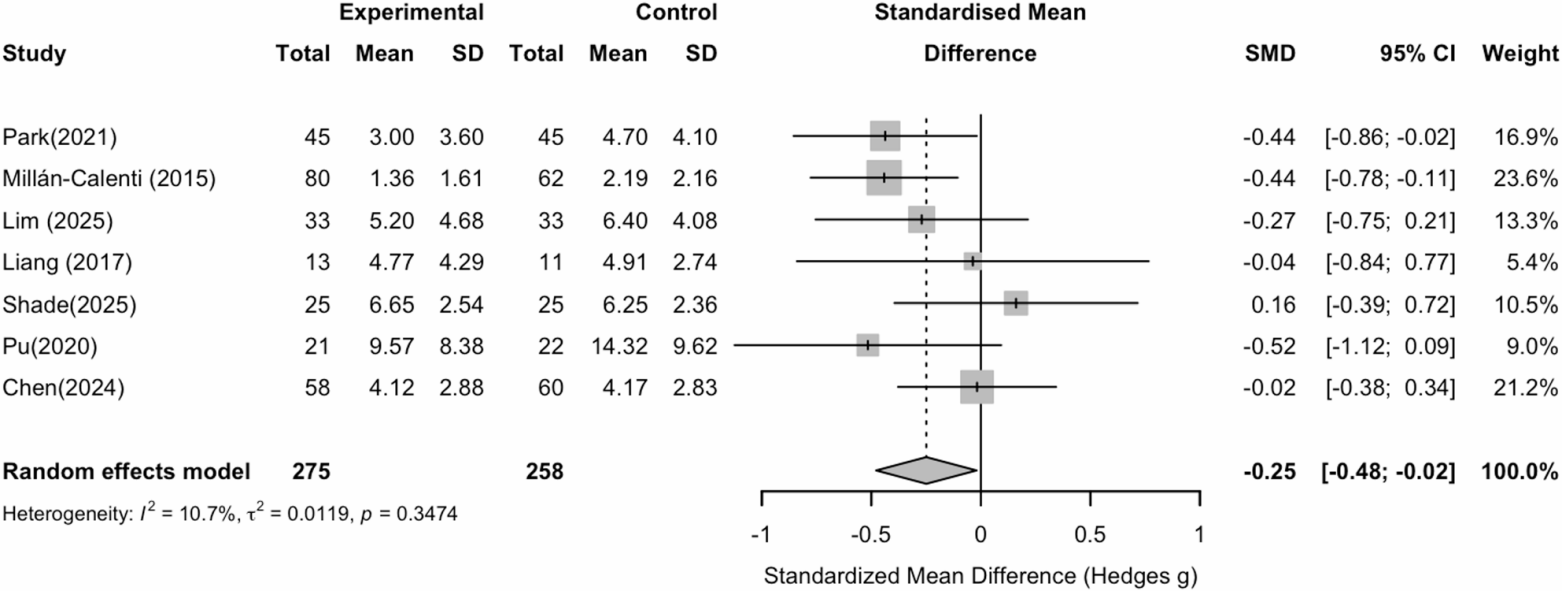
Effects of Artificial intelligence-based conversational and socially assistive agent interventions on loneliness in older adults

Seven randomized controlled trials were included in the analysis of depression outcomes. The sample comprised 275 participants in the intervention groups and 258 participants in the control groups. Artificial intelligence-based conversational and socially assistive agent interventions were associated with a statistically significant reduction in depressive symptoms compared with control conditions (Hedges’ g = -0.25, 95% CI -0.48 to -0.02). The pooled effect size was small in magnitude, with low between-study heterogeneity observed across trials (I² = 10.7%). The forest plot summarizing these randomized controlled trials is presented in Fig. 4.

**Fig. 5:**
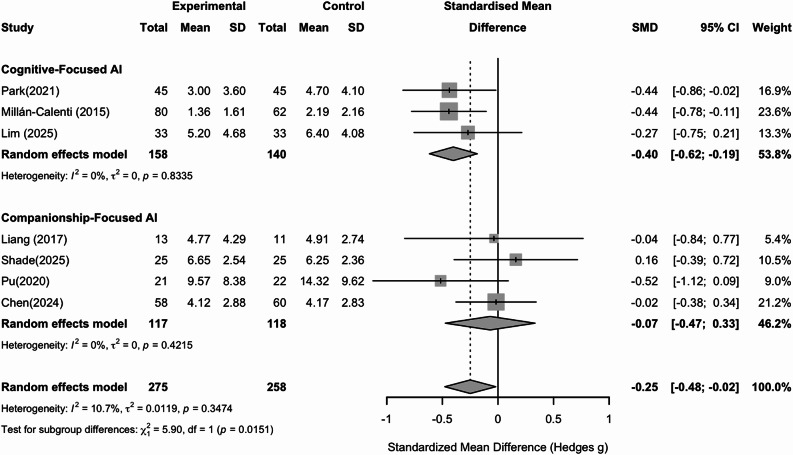
Subgroup analyses of the efficacy of AI intervention orientation type (Cognitive-focused AI vs. companionship-focused AI) for depressive symptoms

A subgroup analysis was further conducted according to AI intervention orientation, namely cognitive-focused AI versus companionship-focused AI, to examine whether intervention orientation influenced intervention efficacy. Cognitive-focused AI interventions were associated with a statistically significant reduction in depressive symptoms (Hedges’ g = -0.40, 95% CI -0.62 to -0.19), and no between-study heterogeneity was observed among the included trials (I² = 0.0%). In contrast, companionship-focused AI interventions did not demonstrate a statistically significant effect (Hedges’ g = -0.07, 95% CI -0.47 to 0.33). Consistently, no heterogeneity was detected across studies evaluating companionship-focused AI interventions (I² = 0.0%). Detailed results of the subgroup analyses are presented in Fig. 5.

**Fig. 6:**
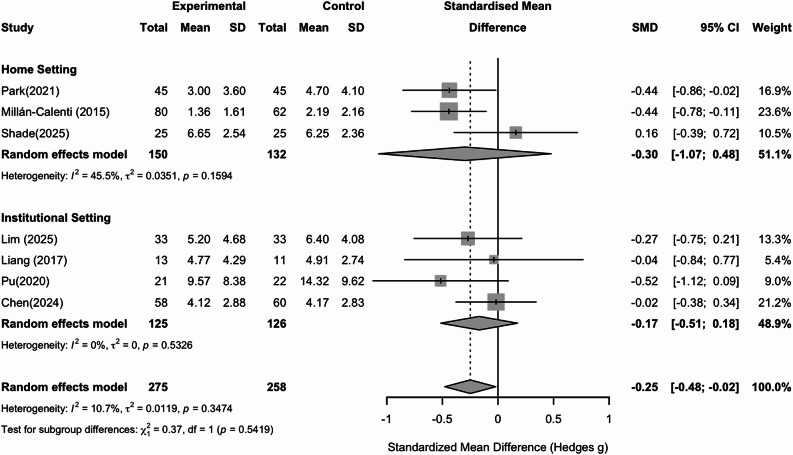
Subgroup analyses of the efficacy of living environment (Home setting vs. Institutional setting) for depressive symptoms

Given the importance of the living environment for mental health in older adults, a subgroup analysis was additionally conducted comparing home-based and institutional settings. Home-based interventions did not demonstrate a statistically significant reduction in depressive symptoms (Hedges’ g = -0.30, 95% CI -1.07 to 0.48). Low to moderate heterogeneity was observed across studies conducted in home settings (I² = 45.5%). Similarly, interventions delivered in institutional settings did not show a statistically significant effect on depressive symptoms (Hedges’ g = -0.17, 95% CI -0.51 to 0.18), and no between-study heterogeneity was detected among the included trials (I² = 0%). The results of the living environment subgroup analyses are presented in Fig. 6.

#### Loneliness

**Fig. 7:**
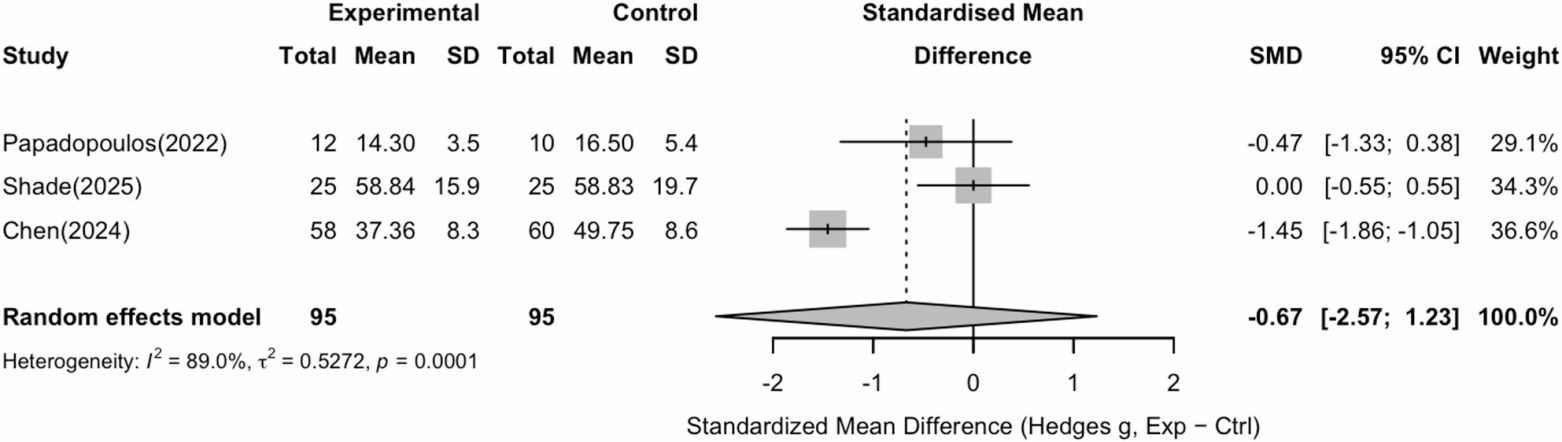
Effects of Artificial intelligence-based conversational and socially assistive agent interventions on depressive symptoms in older adults

The meta-analysis included three randomized controlled trials examining the effects of Artificial intelligence-based conversational and socially assistive agent interventions on loneliness in older adults. The sample comprised 95 participants in the intervention groups and 95 participants in the control groups. AI agent interventions were not associated with a statistically significant reduction in loneliness compared with control conditions (Hedges’ g = -0.67, 95% CI -2.57 to 1.23). Substantial between-study heterogeneity was observed (I² = 89%). Owing to the limited number of included studies, further subgroup analyses to explore sources of heterogeneity were not feasible. The forest plot summarizing these randomized controlled trials is presented in Fig. 7.

## Discussion

This systematic review and meta-analysis synthesized available evidence on the effects of Artificial intelligence-based conversational and socially assistive agent interventions on mental health outcomes in older adults. A total of eight randomized controlled trials comprising 611 participants from eight countries were included, evaluating the effects of artificial intelligence-based or robot-assisted interventions on depressive symptoms and loneliness. Overall, Artificial intelligence-based conversational and socially assistive agent interventions were associated with a statistically significant reduction in depressive symptoms compared with control conditions, whereas no statistically significant effect was observed for loneliness outcomes. Taken together, these findings provide quantitative and evidence-based insights into both the potential benefits and current limitations of AI interventions for older adults, and underscore important directions for future research and clinical application.

### Depression

The present meta-analysis demonstrated that Artificial intelligence-based conversational and socially assistive agent interventions were associated with a statistically significant reduction in depressive symptoms among older adults. This finding is consistent with previous evidence indicating that older adults, including community-dwelling individuals and those experiencing social isolation or subclinical depressive symptoms, may benefit from structured digital interventions delivered over defined intervention periods [27]. Taken together, these findings support the potential of digital interventions as a scalable and accessible strategy for addressing depression in older populations.

A plausible explanation for this effect is that digital interventions may reduce depression related risks by promoting behavioral activation, enhancing daily structure, and encouraging engagement in meaningful activities [28]. Furthermore, regular interaction with digital systems may strengthen perceived social support and self-regulatory capacity, thereby improving self-efficacy and perceived control [29]. These psychosocial mechanisms have been consistently linked to reductions in depressive symptoms among older adults.

Subgroup analyses further suggested that cognitive-focused artificial intelligence interventions were particularly effective in reducing depressive symptoms in older adults. This finding is in line with existing literature indicating that cognitive and behavioral approaches are well-suited for the treatment of depression in later life [30, 31]. Prior research has shown that digitally delivered cognitive behavioral therapy and behavioral activation interventions can yield significant improvements in depressive symptoms among older adults when implemented within structured intervention frameworks [32].

In contrast, companionship-focused artificial intelligence interventions did not exhibit a statistically significant effect on depressive symptoms in the present meta-analysis. One possible explanation is that companionship-focused approaches rely heavily on emotional attunement and empathic responsiveness, capacities that remain challenging for current artificial intelligence systems to fully replicate [33]. Previous research suggests that although companionship-focused chatbots may simulate empathic language, older adults may be particularly sensitive to perceived artificiality or limited relational depth, which could constrain therapeutic engagement and reduce intervention effectiveness [34].

### Loneliness

The present meta-analysis did not observe a statistically significant effect of artificial intelligence-based interventions on loneliness among older adults. This finding aligns with previous evidence suggesting that loneliness in older populations is a complex and multifaceted phenomenon, which may be less responsive to short-term digital interventions [35]. Although AI interventions are designed to simulate social interaction and provide conversational companionship, current evidence indicates that such interactions alone may be insufficient to reduce loneliness in older adults [36].

One possible explanation for this finding is that loneliness is closely associated with broader social conditions, such as reduced social networks, limited social participation, and loss of meaningful interpersonal roles [37]. In older adults, loneliness often arises in the context of bereavement, retirement, or living alone, factors that extend beyond the reach of individual-level psychological interventions [38]. While AI chatbots may provide momentary companionship or emotional support, they have limited capacity to alter the broader social environments that contribute to persistent loneliness.

Furthermore, loneliness in older adults is strongly linked to perceived relational quality rather than the mere presence of interaction [39]. Meaningful social connections typically require mutuality and emotional reciprocity, which remain challenging for AI systems to replicate [40]. Research suggests that although conversational agents can engage users through responsive dialogue, older adults may perceive these interactions as lacking depth or empathy, thereby limiting their impact on intervention efficacy [41]. In addition, older adults may differentiate clearly between human relationships and artificial agents, which could constrain the extent to which AI systems’ interactions influence self-reported loneliness [42].

### Clinical implications

The findings of this review have several implications for clinical practice. AI-based conversational and socially assistive agents demonstrated a potential benefit in reducing depressive symptoms among older adults, suggesting that such technologies may serve as supportive tools within mental health care settings. These systems may be particularly useful for enhancing engagement, providing structured cognitive stimulation, or offering emotional support in contexts where human resources are limited.

In contrast, no statistically significant effect was observed for loneliness. Loneliness represents a complex and relational construct influenced by broader social and environmental factors. Short-term AI-based interventions may provide momentary interaction or companionship but may be insufficient to modify the structural determinants of persistent loneliness. From a precision health perspective, the effectiveness of AI interventions may depend on aligning specific technological functionalities with individual needs and contextual factors.

Clinicians and care providers should therefore consider AI-based agents as complementary tools rather than substitutes for human relationships. While these technologies may support emotional well-being and cognitive engagement, interventions targeting loneliness may require integrated approaches that incorporate social, community, and interpersonal components.

### Limitations and future direction

Several limitations of the present meta-analysis warrant consideration. First, the number of eligible randomized controlled trials specifically targeting older adults remains limited. Although stringent inclusion criteria were applied to ensure methodological rigor, this resulted in a relatively small number of studies being included in the quantitative synthesis.

Second, heterogeneity was observed across studies in participant characteristics, intervention duration, and outcome measurement. Substantial heterogeneity was particularly evident in the loneliness analysis (I² = 89%). However, sensitivity analyses demonstrated that exclusion of individual studies markedly reduced heterogeneity estimates while the direction and statistical non-significance of the pooled effect remained unchanged. These findings suggest that, although heterogeneity was present, the overall conclusion regarding loneliness outcomes was not driven by any single study and should be interpreted with appropriate caution.

Third, the included interventions demonstrated variability in reported AI-related functionalities and technological implementation. Although all studies met the predefined eligibility criteria and excluded purely rule-based systems, differences in the extent and reporting of adaptive or interactive features may have influenced intervention effects. The limited number of studies and heterogeneous reporting of AI mechanisms precluded formal subgroup analyses based on specific technological characteristics.

Fourth, the effectiveness of AI agent interventions may be influenced by users’ familiarity with and acceptance of digital technologies. Older adults often require an adaptation or learning period when interacting with AI systems, and initial unfamiliarity may reduce engagement or limit early intervention effects. Moreover, AI-based interventions are highly dependent on technological design and implementation quality. Variations in responsiveness, conversational coherence, personalization, and error handling can substantially influence user experience and therapeutic engagement. Future research should prioritize conducting more rigorous randomized controlled trials to explore optimal intervention content and duration, and to better align AI-based interventions with the real-world needs of older adults.

## Conclusion

Artificial intelligence-based conversational and socially assistive agent interventions were associated with a statistically significant reduction in depressive symptoms among older adults, whereas no significant improvement was observed for loneliness outcomes. Subgroup analyses suggested that cognitive-focused AI interventions produced greater reductions in depressive symptoms compared with companionship-focused approaches, while intervention effects did not differ significantly between home-based and institutional settings. Overall, although AI-driven interventions may represent a promising adjunctive strategy for supporting mental health in older adults, their effectiveness appears to be contingent on intervention orientation and implementation characteristics. Consequently, the selection and application of Artificial intelligence-based conversational and socially assistive agents should be carefully tailored to individual needs, cognitive status, and contextual factors, and guided by rigorous, criteria-based evaluation.

## Supplementary Information

Supplementary Material 1.

Supplementary Material 2.

## Data Availability

The datasets analyzed during the current study were extracted from previously published studies and are available from the corresponding author upon reasonable request.

## Abbreviations

AI: Artificial Intelligence
RCTs: Randomized controlled trials
RoB 2: Risk of Bias 2
GRADE: Grades of Recommendation, Assessment, Development and Evaluation
