# Supplementary material for: Effectiveness of AI-based conversational and socially assistive agents in older adults: a systematic review and meta-analysis
Source: BMC Geriatr. 2026 May 7;26:887. doi: 10.1186/s12877-026-07418-6 (PMC13321516; doi:10.1186/s12877-026-07418-6)
Supplement: Supplementary file 1 — Supplementary Material 1. [file 12877_2026_7418_MOESM1_ESM.docx]

**Appendix 1：search strategy**

A systematic literature search was conducted across PubMed, Embase, Web of Science, the Cochrane Library, and PsycArticles, covering all records published from database inception to November 15, 2025.

| **Database** | **Retrieval strategy** |
| --- | --- |
| **Cochranne （n=747)** | (older adults OR elderly OR aging OR older people ) AND ( artificial intelligence OR AI OR chatbot* OR conversational agent* OR mental health chatbot* ) AND ( depression OR depressive symptoms OR loneliness OR social isolation OR mental health OR emotional well-being) |
| **Embase**  **(n=118)** | ('aged'/exp OR 'elderly'/exp OR 'older adult*':ti,ab OR elderly:ti,ab OR aging:ti,ab) AND ('artificial intelligence'/exp OR 'chatbot'/exp OR chatbot*:ti,ab OR 'conversational agent*':ti,ab OR 'mental health chatbot*':ti,ab) AND ('depression'/exp OR 'loneliness'/exp OR depression:ti,ab OR 'depressive symptom*':ti,ab OR loneliness:ti,ab OR 'social isolation':ti,ab OR 'emotional well-being':ti,ab) ) |
| **Web of Science**  **(n=1588)** | ("older adults" OR elderly OR aging OR "older people") AND ("artificial intelligence" OR AI OR chatbot OR "conversational agent") AND (depression OR loneliness OR "social isolation" OR "mental health") |
| **PubMed**  **(n=1013)** | ( ("Aged"[Mesh] OR older adults[Title/Abstract] OR elderly[Title/Abstract] OR aging[Title/Abstract] OR older people[Title/Abstract]) ) AND ( ("Artificial Intelligence"[Mesh] OR chatbot*[Title/Abstract] OR conversational agent*[Title/Abstract] OR AI[Title/Abstract] OR mental health chatbot*[Title/Abstract]) ) AND ( ("Depression"[Mesh] OR "Loneliness"[Mesh] OR depression[Title/Abstract] OR depressive symptom*[Title/Abstract] OR loneliness[Title/Abstract] OR social isolation[Title/Abstract] OR mental health[Title/Abstract] OR emotional well-being[Title/Abstract]) ) |
| **PsycArticles**  **(n=25)** | Any Field: DE "Aging" OR Any Field: DE "Older Adults" OR Any Field: elderly OR Any Field: "older adults")) AND (Any Field: DE "Artificial Intelligence" OR Any Field: chatbot* OR Any Field: "conversational agent*" OR Any Field: "mental health chatbot*") AND (Any Field: DE "Depression" OR Any Field: DE "Loneliness" OR Any Field: depression OR Any Field: depressive symptoms OR Any Field: loneliness OR Any Field: social isolation OR Any Field: emotional well-being |
