# Supplementary figures and images for: Effectiveness of AI-based conversational and socially assistive agents in older adults: a systematic review and meta-analysis
Source: BMC Geriatr. 2026 May 7;26:887. doi: 10.1186/s12877-026-07418-6 (PMC13321516; doi:10.1186/s12877-026-07418-6)

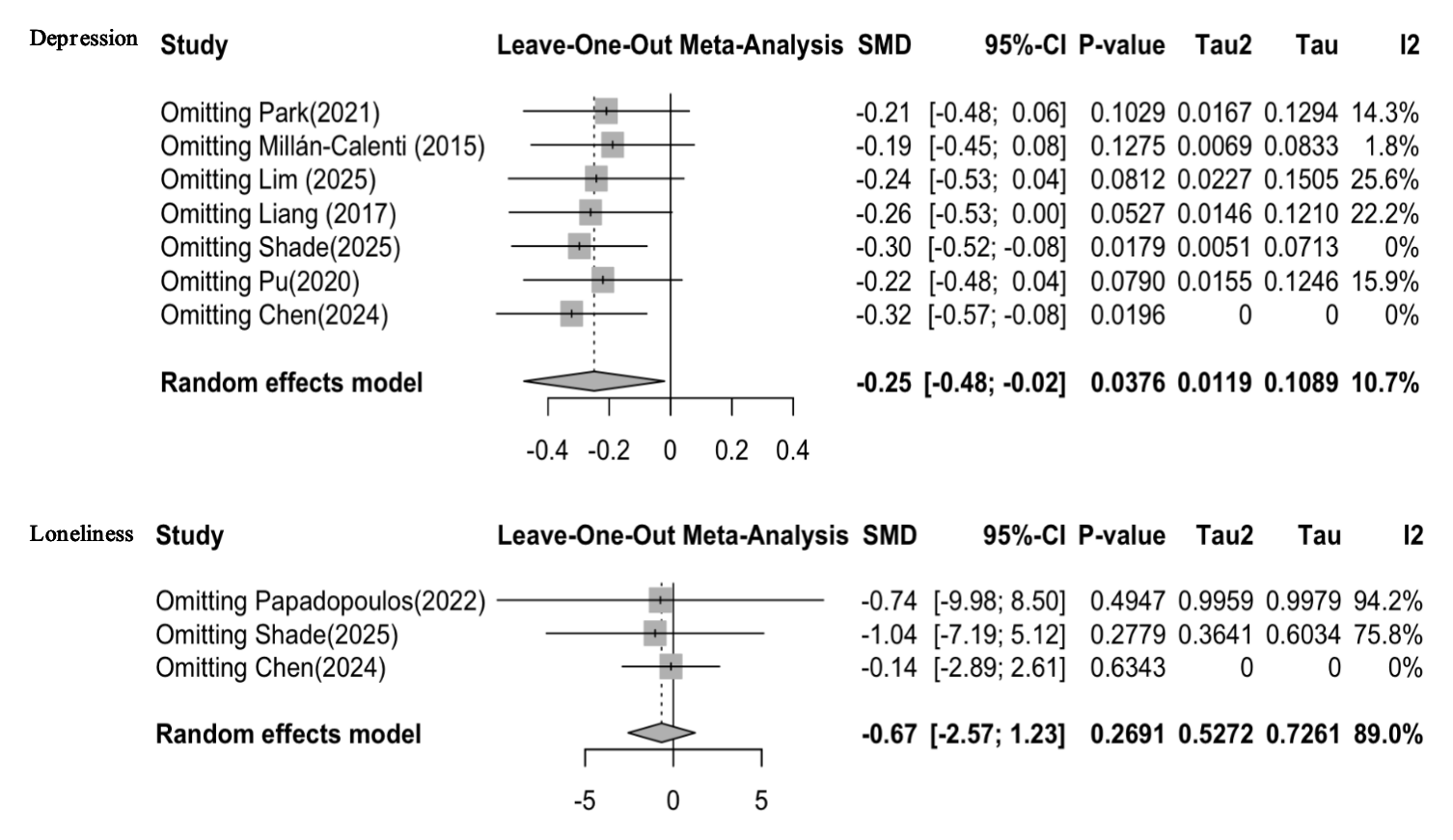

Supplement: Supplementary file 2 — Supplementary Material 2. [file 12877_2026_7418_MOESM2_ESM.tif]
